# Lying in psychiatry: A review

**DOI:** 10.1192/j.eurpsy.2021.1271

**Published:** 2021-08-13

**Authors:** M.T. Valadas, R. Mota Freitas

**Affiliations:** 1 Serviço De Psiquiatria, Unidade Local de Saúde do Baixo Alentejo, Beja, Portugal; 2 Departamento De Psiquiatria E Saúde Mental, Hospital do Espírito Santo de Évora, Évora, Portugal

**Keywords:** Lying

## Abstract

**Introduction:**

Lying can be defined as stating a deliberate falsehood with the intent to deceive. It is part of our everyday life but it can be pathological, without motivation and a symptom of psychiatric illness. Although pathological lying has been debated for a century, it remains a controversial issue in Psychiatry.

**Objectives:**

We aim to perform a review regarding pathological lying and related issues.

**Methods:**

We performed an updated review in the PubMed database and GoogleScholar using the terms “pathological lying”, “compulsive lying”, “mythomania” and “pseudologia fantastica”. The included articles were selected by title and abstract. We also consulted reference textbooks.

**Results:**

We described the difference between normal and pathological lying and debated the different types of pathological lying, such as compulsive lying, mythomania and pseudologia fantastica.

**Conclusions:**

Recognizing lying is crucial for a skilled patient interview and distinguishing between pathological and non pathological lying may be decisive for an accurate differential diagnosis.

